# Application of near-infrared spectroscopy for the nondestructive analysis of wheat flour: A review

**DOI:** 10.1016/j.crfs.2022.08.006

**Published:** 2022-08-23

**Authors:** Shun Zhang, Shuliang Liu, Li Shen, Shujuan Chen, Li He, Aiping Liu

**Affiliations:** College of Food Science, Sichuan Agricultural University, Ya'an, Sichuan, 625014, PR China

**Keywords:** Near-infrared spectroscopy, Nondestructive analysis, Wheat flour, Quality, Safety

## Abstract

The quality and safety of wheat flour are of public concern since they are related to the quality of flour products and human health. Therefore, efficient and convenient analytical techniques are needed for the quality and safety controls of wheat flour. Near-infrared (NIR) spectroscopy has become an ideal technique for assessing the quality and safety of wheat flour, as it is a rapid, efficient and nondestructive method. The application of NIR spectroscopy in the quality and safety analysis of wheat flour is addressed in this review. First, we briefly summarize the basic knowledge of NIR spectroscopy and chemometrics. Then, recent advances in the application of NIR spectroscopy for chemical composition, technological parameters, and safety analysis are presented. Finally, the potential of NIR spectroscopy is discussed. Combined with chemometric methods, NIR spectroscopy has been used to detect chemical composition, technological parameters, deoxynivalenol, adulterants and additives of wheat flour. Furthermore, NIR spectroscopy has shown great potential for the rapid and online analysis of the quality and safety of wheat flour. It is anticipated that the current review will serve as a reference for the future analysis of wheat flour by NIR spectroscopy to ensure the quality and safety of flour products.

## Highlights

• NIR spectroscopy is an ideal technique for analysis of wheat flour due to its rapid and nondestructive nature.
• Use of NIR spectroscopy for chemical composition, technological parameters, and safety analysis.
• Online and handheld NIR spectrometers for wheat flour detection are the future trends.

## Graphical abstract

**Figure:**
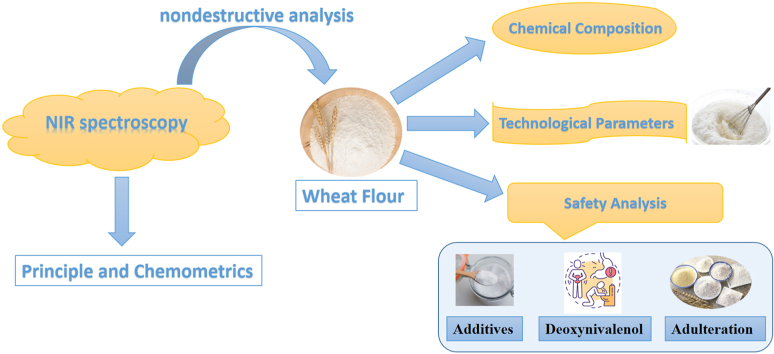

## 1 Introduction

Wheat flour is a powdered product made from wheat kernel and mainly used for manufacturing various bakery and pasta products, such as breads, cakes, biscuits and noodles (Zareef et al., 2021). As one of important consumable raw materials in our daily lives, wheat flour provides numerous nutrients, such as carbohydrates, protein, and minerals. However, the quality and safety of wheat flour products are sometimes challenged by the inferior quality parameters and adulteration, which cannot be easily detected and pose risks to human health. Consequently, there is an urgent need to develop a rapid, labor-saving and efficient analytical method for the quality and safety monitoring of wheat flour.

The main quality parameters of wheat flour include its chemical composition, which is related to the moisture, protein, ash, and wet gluten contents of the flour, and technological parameters, such as the sedimentation value, falling number and rheological properties of wheat flour dough. Conventional methods available for the quality and safety assessment of wheat flour are listed in Table 1. Although these methods have good precision, most of them are laborious and time-consuming. Therefore, the quality and safety of wheat flour cannot be monitored quickly and efficiently.

**Table 1:** Conventional methods for quality and safety parameters of wheat flour.

Parameter	Methods	Reference
Protein	Kjeldahl method AACC 46-12, ICC 105-2, Dumas combustion AACC 46-30, ICC 167	AACC (2002); ICC (1994); Sezer et al. (2016)
Moisture	Gravimetric method, AACC 44-15A, ICC 110-1	AACC (2002); ICC (1994); Takeuti et al. (2016)
Ash	Burning method, AACC 8-01, ICC 104-1	AACC (2002); Czaja et al. (2020); ICC (1994)
Rheological properties	Mixolab, Farinograph and Extensograph AACC54-21, AACC 56–61A, ICC 116-1	AACC (2002); ICC (1994); Parenti et al. (2021)
Wet gluten content	Automatic glutomatic machine method AACC 38-12A, ICC 137-1	AACC (2002); Barakat et al. (2020); ICC (1994)
Falling number	Hagberg-Perten method, AACC 56–81B, ICC 107-1	AACC (2002); Guan et al. (2020); ICC (1994)
Additives	High-performance liquid chromatography; X-ray diffraction	F. Chen et al. (2021); Kim et al. (2022)
Deoxynivalenol	Liquid chromatography-mass spectrometry; enzyme-linked immunosorbent assay; thin-layer chromatography method AACC 45-41	AACC (2002); Li et al. (2021); Okuma et al. (2018)

Recently, near-infrared (NIR) spectroscopy, as a reliable tool in agricultural and food industry analysis, has been widely used in the daily inspection of wheat flour (Delwiche, 1998; Porep et al., 2015). NIR spectroscopy, which has the advantages of fast, easy operation, high efficiency and nondestructive measurement, can be used for qualitative and quantitative analysis of basic components in samples and the detection of adulterated samples. The applications of NIR spectroscopy in the quality and safety evaluation of tea products (Lin and Sun, 2020), grain (Caporaso et al., 2018), fruits and vegetables (Nicolai et al., 2007), oilseeds and edible oils (Li et al., 2020) have been reviewed. Although reviews on wheat-based products (Badaró et al., 2022) and wheat flour (Du et al., 2022) were recently reported, respectively, the safety analysis of wheat flour was not fully illustrated. Therefore, this review first summarizes the basic knowledge of NIR spectroscopy. Then, recent advances in the quality and safety evaluation of wheat flour by NIR spectroscopy, including the analysis of basic nutritional components, technological parameters and safety, are reviewed. In addition, future trends and challenges of NIR spectroscopy are presented.

## 2 Principle of NIR spectroscopy and chemometrics

As defined by the American Society for Testing and Materials (ASTM), near-infrared light is part of the electromagnetic spectrum in the range of 780–2500 nm, which is between the visible light and mid-infrared light spectrum (Pasquini, 2018). NIR spectroscopy is a technique that applies the NIR portion of the electromagnetic spectrum and can provide complex structural information related to the vibration behavior of chemical bonds (Kamal and Karoui, 2015). NIR spectra present the overtones and combination of hydrogen-containing C–H, O–H and N–H groups, which are the primary structural components of organic compounds, such as water, lipids and proteins (Futami et al., 2016; Wang et al., 2019). In other words, NIR spectroscopy is a technique in which the instrument emits wavelengths across the entire near-infrared spectrum that penetrate the sample. Some wavelengths are absorbed through the activation of specific chemical bonds within the sample, while the remaining wavelengths are transmitted or reflected back to the instrument, forming the resulting spectrum (Johnson, 2020). Combined with chemometrics, the spectral data collected by the NIR spectrometer are further utilized for qualitative and/or quantitative analysis of products. The whole measurement process of NIR spectroscopy generally includes the following steps: (i) spectral data acquisition; (ii) data preprocessing; (iii) use of a set of samples with known analytical concentrations to establish a calibration model; (iv) validation of the model; and (v) prediction or characterization of the unknown samples (Cen and He, 2007).

The NIR spectrometer is the hardware for near-infrared analysis and is mainly composed of a light source, a beam splitting system, a sample detector, an optical detector, and a data processing and analysis system (Cen and He, 2007). Based on the spectroscopic system, NIR spectrometers can be divided into four types: filter type, dispersion type, interference type and acousto-optical tunable filter type (Pasquini, 2018). In terms of applications, NIR spectrometers can be divided into laboratory spectrometers, portable spectrometers and online spectrometers. In the past ten years, different types of NIR spectrometers have developed rapidly, such as visible/shortwave near-infrared spectrometers (Vis/SW-NIR) (Barragan et al., 2021), miniaturized and handheld near-infrared spectrometers (Mcgrath et al., 2020), near-infrared hyperspectral imaging (NIR-HSI), which integrates sample spectra and images (Khamsopha et al., 2021).

Compared with traditional methods for quality and safety analysis of wheat flour, the main technical features of NIR spectroscopy include fast analysis speed, convenient operation, simultaneous determination and nondestructive sampling. In analytical processes, the combination of NIR and chemometrics is essential (Qu et al., 2015). Chemometrics is the multivariate data analysis application that uses mathematical and statistical methods to systematically study the connotation of chemical measurement values (Yin et al., 2021). The application of chemometrics in NIR spectral analysis includes three aspects: (i) spectral data pretreatment; (ii) establishment of a calibration model for quantitative and qualitative analysis; and (iii) model transfer (Cortés et al., 2019). The pretreatment of the original spectral data can be used to remove interference information and improve the modeling effect. The main pretreatment techniques are smoothing, normalization, wavelet transform, multiplicative scatter correction, orthogonal signal correction, standard normal variable (SNV), first derivative or second derivative, direct quadrature signal correction and straight line subtraction (Arslan et al., 2021). The chemometric algorithms used for modeling include principal component analysis (PCA), artificial neural networks (ANNs), partial least squares (PLS), partial least squares discriminant analysis, linear discriminant analysis (LDA), multiple linear regression, support vector machines (SVMs), radial basis functions (RBFs), back propagation (BP), random forests (RFs), extreme learning machine (ELM), soft independent modeling of class analogy, and cluster analysis (CA) (Dankowska and Kowalewski, 2019; Granato et al., 2018; Shahbazi and Esfahanian, 2019; Zhang et al., 2020). To obtain a stable and robust model, evaluation of the final model is critical. The correlation coefficient (R), coefficient of determination (R^2^) and correlation coefficient for prediction (R_p_) are often used to evaluate the performance of built models. The best models typically have the highest R and R^2^ or sometimes R_p_ while having a lower root mean square error of prediction (RMSEP) and root mean square error of cross-validation (RMSECV) (Minas et al., 2021). Additionally, residual predicted deviation (RPD) is used to evaluate the stability of the model, and a higher RPD indicates a better predictive performance (Kutsanedzie et al., 2018).

## 3 Chemical composition analysis of wheat flour

The main components of wheat flour analyzed include moisture, protein, ash, and some functional substances, which are closely related to its nutritional quality and processing properties. The hydrogen-containing groups (O–H, N–H, C–H, and S–H bonds) in each component of wheat flour have characteristic absorption peaks in the near-infrared spectral region, which is the foundation for the detection of chemical constituents of wheat flour by NIR spectroscopy (Wadood et al., 2019). NIR spectroscopy combined with chemometrics has been successfully applied to analyze chemical composition of wheat flour (Wadood et al., 2019).

### 3.1 Moisture

Moisture is an important quality parameter for wheat flour storage and processing, and moisture content is typically less than 14.5% (Khalid et al., 2017). PLS regression (PLSR) is the most commonly used regression algorithm for predicting moisture content. A moisture content PLSR model was established based on 120 wheat flour samples using NIR spectroscopy, and the R, R^2^, RMSEP, RMSECV and RPD were 0.92, 0.85, 0.27, 0.47, and 2.43, respectively (Kahrıman and Egesel, 2011). The developed calibration models were successfully used to estimate the moisture content of wheat flour. Dong and Sun (2013) selected characteristic bands of 4000–4896 cm^−1^ and 5504-6704 cm^−1^ that related to moisture by interval partial least squares (iPLS), and applied PLSR to establish the model, which showed R_p_ and RMSEP of 0.99 and 0.088, respectively.

The handheld NIR spectrometer is also suitable for fast and quantitative determination of moisture in wheat flour. The moisture content in wheat flour samples was evaluated quickly and quantitatively using a linear variable filter-based Viavi MicroNIR 1700 spectrometer by X. Chen et al. (2021). The established PLS calibration model yielded an R^2^ of 0.8367, and the RMSEP and RMSECV were 0.32 and 0.35, respectively. In a study by Sun et al. (2018), the NIR spectral data of wheat flour were collected and analyzed using a MicroNIR-2200 NIR spectrometer, and then the PLS model was utilized to detect the moisture content in wheat flour. The model performed well and the R^2^, RMSEP and RMSECV were 0.929, 0.154, and 0.125, respectively. In addition, Mutlu et al. (2011) analyzed the moisture content of wheat flour using the ANN, and the resulting R^2^ of the moisture content was 0.920.

### 3.2 Protein

The quality and quantity (normal variation ranges 8–16%) of wheat flour protein affect wheat flour dough properties and the quality of the final products (Gabriel et al., 2017; Korkmaz et al., 2021). Kahrıman and Egesel (2011) utilized the first derivative and SNV to preprocess the NIR spectrum and then established a PLS model to determine the protein content in wheat flour with an R^2^ of 0.81 and an RMSEP of 0.58. Jin et al. (2011) improved the prediction effect of protein content model by applying the second derivative to preprocess the spectrum, and an R^2^ of 0.937 and an SEP of 0.492 were obtained. A PLS model of the protein content was established byX. Chen et al., 2021 using a handheld NIR spectrometer based on the Fourier transform technique, and the results showed excellent performance with an R^2^ of 0.9624 and an RMSEP of 0.22. Generally, support vector regression (SVR) is superior to the PLS and ANN methods in modeling NIR data and the synergy interval has a strong ability to select appropriate variables during model building (Lin et al., 2014). In the study by Chen et al., 2017a, Chen et al., 2017b, the effects of spectral pretreatment and synergy interval on the model performance were researched, and the optimal protein model was established. The results showed that the R^2^ and RMSEP were 0.906 and 0.425, respectively. Meanwhile, the results revealed that the models based on the original spectra are generally unacceptable, but they can be substantially improved by applying a proper spectral pretreatment approach.

### 3.3 Ash

Ash contains mineral elements such as calcium, magnesium, phosphorus, and potassium. The ash content indicates the milling degree of wheat flour and serves as an important indicator of the wheat flour's quality and usage (Czaja et al., 2020). In recent years, many studies have confirmed the feasibility of the quantitative determination of wheat flour ash content by NIR spectroscopy (Gao et al., 2021). Dong and Sun (2013) built an ash model for NIR spectroscopy and used interval PLS as the characteristic band selection method ranging from 4000 to 5500 cm^−1^ and 6708-7304 cm^−1^. The predictive ability of ash content models was improved with an R_p_ of 0.911 and an RMSEP of 0.019 using the characteristic bands. A PLS calibration model of ash content was established after pretreatment by the first derivative and SNV within the wavelength range of 908–1676 nm; R^2^ and RMSEP values of 0.9431 and 0.06, respectively, were achieved (X. Chen et al., 2021).

### 3.4 Wet gluten

Wet gluten is a viscoelastic soft gelatinous substance that remains after the starch, water-soluble carbohydrates, fats and other ingredients in the dough formed by mixing wheat flour with water are washed with water. It is mainly composed of gliadin and glutenin, and its content affects the quality and technological properties of wheat flour baked products (Chandi and Seetharaman, 2012). A PLS model was built by applying the spectral information in the 1200–2400 nm range, and the prediction effect of the gluten content of wheat flour was good, with an R^2^ of 0.88 (Kahrıman and Egesel, 2011). Baslar and Ertugay (2011) used NIR spectroscopy to establish wet gluten content correction models for 120 kinds of bread wheat flour from different regions and obtained good results, with R and SEP values of 0.976 and 1.36, respectively. Albanell et al. (2012) established a wet gluten content prediction model using modified PLSR correction, and the best model for wheat flour was obtained, with an R^2^ of 0.985. In the study conducted by Chen et al., 2017a, Chen et al., 2017b, three spectral intervals of 10719.02–9839.59 cm^−1^, 5396.15–4516.72 cm^−1^ and 4509.01–3629.58 cm^−1^ were selected. Standard normal variate, first derivative and support vector regression were subsequently used to establish the wet gluten content siSVR model. As a result, the R^2^, RMSEP and standard deviation ratio values of the optimal model for wet gluten content were 0.850, 1.024 and 2.482, respectively. Furthermore, the feasibility of rapid quantitative analysis of wet gluten of wheat flour samples with handheld NIR spectrometers based on a linear variable filter was investigated by X. Chen et al. (2021), and the model achieved an R^2^_p_ and an RMSEP of 0.8585 and 0.66, respectively.

### 3.5 Other chemical components

In addition to the main chemical composition attributes, the determination of total phenolic, mineral element, and fiber contents as well as free fatty acids of wheat flour were also studied by NIR spectroscopy. Free fatty acids is one of the important indices used to evaluate wheat flour quality during storage. A portable NIR spectroscopy system was developed for the quantitative detection of free fatty acids content in wheat flour during storage by Jiang et al. (2020). Standard normal variate (SNV) and variable combination population analysis (VCPA) methods were used to pretreat the spectral data, and extreme learning machine (ELM) was employed to construct quantitative detection models based on different characteristic wavelength variables to achieve quantitative detection of free fatty acids. The ELM models showed good prediction accuracy and stability when predicting independent samples in the validation set, and the mean R^2^_p_ when using the ELM models was above 0.96. Moreover, a PLS model of oleic acid content in wheat flour during long-term storage was established by Lancelot et al. (2021).

Phenolic compounds contribute greatly to the health benefits of whole wheat products. Tian et al. (2021) presented a novel application of NIR spectroscopy for total phenolic content prediction in whole wheat flour. The optimal regression model demonstrated R^2^ values of 0.92 and 0.90 for the calibration and validation sets, respectively, and an RPD value of 3.4.

Fiber in wheat flour can increase its nutritional value, but also affect its functional properties. Therefore, it is important to detect the fiber content and distribution in wheat flour. The amount of fiber added to semolina and its distribution were investigated via NIR spectroscopy and hyperspectral imaging (NIR-HSI) by Badaro et al. (2019), and the results of PLSR models showed that the R^2^_p_ was between 0.85 and 0.98, and the RMSEP was between 0.5 and 1%.

In addition, NIR spectroscopy coupled with PLS was successfully applied to establish a model for the rapid prediction of mineral elements (calcium, phosphorus and potassium) contents in wheat flour samples. The R^2^ values of the best models for calcium, phosphorus and potassium were 0.7907, 0.9777, and 0.9777, respectively; the RMSEP values were 5.35, 15.3, and 18.9, respectively; and the RPD were 2.19, 6.71, and 6.84, respectively (Gao et al., 2021). Although the R^2^ of the calcium model is low, NIR can still predict the calcium content of wheat flour, and NIR also has excellent predictive performance for the phosphorus and potassium content in wheat flour.

## 4 Technological parameters analysis of wheat flour

It is known that NIR spectroscopy can be used to predict wheat flour technological parameters since abundant changes in the technological parameters of wheat flour are related to the chemical variation of its components (Kaddour and Cuq, 2011; Lancelot et al., 2021). Zeleny sedimentation value can reflect the quality and quantity of gluten protein in wheat flour and predict the rheological properties of dough. NIR spectroscopy was used to develop the calibration models for the Zeleny sedimentation test of bread wheat flours collected from different regions of Turkey by Baslar and Ertugay (2011). Reasonable model results were obtained for the Zeleny sedimentation test with an R of 0.924 and a SEP of 3.74. Mutlu et al. (2011) predicted the Zeleny sedimentation value and the water absorption of wheat flour by using NIR spectroscopy combined with an ANN. The prediction accuracy was good, with R^2^ values of 0.917 and 0.832, respectively.

In the study conducted by Chen et al., 2017a, Chen et al., 2017b, the PLS models of near-infrared based on different spectral pretreatment methods were adopted to predict water absorption, dough development time, and dough stability, but the performance was unsatisfactory, with a R and an RMSEP for each parameter of 0.7 and 1.560, 0.73 and 1.065, 0.79 and 1.090, respectively. X. Chen et al. (2021) used a handheld NIR spectrometer to investigate the sedimentation value of wheat flour, and the R and RMSEP of the calibration model were 0.8185 and 2.12, respectively. In the study conducted by Lancelot et al. (2021), a good prediction was observed for the Hagberg falling number, swelling index and solvent retention capacity (SRC), which reflected α-amylase, amylose-amylopectin ratio, and gluten viscoelasticity, respectively, but unsatisfactory results were obtained for the farinograph parameters. Additional applications of NIR in detecting the technological parameters of wheat flour are summarized in Table 2.

**Table 2:** Applications of NIR spectroscopy in the detecting of technological parameters of wheat flour.

Parameters	Spectral range	Data analysis	Accuracy/Performance	Reference
Sedimentation	400–2500 nm	MPLS	R^2^ = 0.6, SEP = 6.5	Jirsa et al. (2007)
Hagberg Falling number	4000–10000 cm^−1^	PLSR	R^2^ = 0.982, RMSEV = 7.550	Lancelot et al. (2021)
Swelling index	4000–10000 cm^−1^	PLSR	R^2^ = 0.874, RMSEV = 0.981	Lancelot et al. (2021)
solvent retention capacity	4000–10000 cm-1	PLSR	R^2^ = 0.862, RMSEV = 0.846	Lancelot et al. (2021)

## 5 Safety analysis of wheat flour

Wheat flour safety issues typically involve chemical additives and undesired contaminants, as well as the adulteration of wheat flour. Generally, the addition of inexpensive substitutes, such as rice, corn or potatoes, can reduce the processing performance and commercial value of wheat flour (Che et al., 2017). And unapproved chemical additives or undesirable compounds in wheat flour may mask its quality, and even affect consumer health (Annalisa et al., 2020). Wheat flour may also be contaminated by other flours and mycotoxins during storage and processing. Currently, the analysis of the safety of wheat flour is a primary concern in the food and agricultural product markets. General chemical methods cannot effectively identify the adulteration of wheat flour due to the similar taste, appearance, and physicochemical properties of other flours. As an excellent measurement tool, NIR spectroscopy has been widely used for the detection of safety in a variety of complex foods (Pereira et al., 2020; Yuan et al., 2020). In terms of wheat flour, NIR spectroscopy has been widely applied to the detection of chemical additives, biological contaminants and the adulterant use of other flours in wheat flour. Besides, the contamination of allergens in wheat flour is also an important safety issue. For example, Zhao et al., 2018a, Zhao et al., 2018b have used the NIR-HSI technique to predict the contamination concentrations of peanut and walnut flour in whole wheat flour. The optimal general multispectral model had promising results, with R^2^_p_ and RMSEP values of 0.987 and 0.373%, respectively. However, there are few studies in this area at present, and this research direction is worthy of attention.

### 5.1 Chemical additives

Chemical additives are used to standardize the quality and process performance of wheat flour and chemical additives used in wheat flour include benzoyl peroxide (BPO), talcum powder, azodicarbonamide, ascorbic acid, emulsifiers, enzymes, etc (Luis et al., 2017; Hu et al., 2018; C. Zhao et al., 2020). Some of these additives should be used within the established limits, while some are prohibited in wheat flour because of the potential for serious adverse effects (Fu et al., 2020; Matina et al., 2011). Meanwhile, some additives or undesirable chemicals may be excessively added to the flour for profit. Therefore, the safety and quality of wheat flour are challenged by chemical additives.

The BPO content in pure wheat flour was determined based on NIR diffuse reflectance spectroscopy by Zhang et al. (2011), and the R^2^_cal_, RMSEC, R^2^_pred_, and RMSEP of the PLS model were 0.8901, 40.85 mg/kg, 0.8865, and 44.69 mg/kg, respectively. Another study on detecting the concentration of benzoyl peroxide in wheat flour was conducted by Sun et al. (2016), who designed prediction models based on NIR reflectance spectroscopy integrated with PLS, BP neural networks, and RBF neural networks separately. The results showed that the RBF neural network model had optimal predictive accuracy and feasibility, with R, RMSEP, and RPD values of 0.9937, 15.5095, and 8.8216, respectively. At the same time, Deng et al. (2019) extracted the optimal wavelengths and then established a competitive adaptive reweighted sampling (CARS) model for talc content in wheat flour by NIR. The verification set R^2^_p_ was 0.998 and the RMSEP was 0.282%, and the detection limit of the model reached 0.5%. Furthermore, the results of the study by Fu et al. (2020) demonstrated that talcum powder and BPO could be effectively discriminated in wheat flour.

In addition, a feasibility study was conducted by Wang et al. (2013) to rapidly test lime and calcium carbonate concentrations in wheat flour samples using NIR with the PLS algorithm. The results indicated that the R^2^ values of lime and calcium carbonate using the PLS algorithm were 99.80% and 96.98%, the RMSEC were 0.19 and 0.34, the RMSECV were 0.26 and 0.75, the RMSEP were 0.63 and 0.44, and the RPD were 8.57 and 5.24, respectively. In terms of azodicarbonamide (ADA) detection, Gao et al. (2016) utilized NIR spectroscopy in combination with RBF to quantitatively detect the content of ADA in wheat flour. The established model presented good prediction indicators, with R, RMSEP, and RPD values of 0.97828, 18.2887 mg/kg, and 4.7621, respectively. The limits of quantitation and detection of the model were 72 and 15 mg/kg, respectively. Recently, the ADA content in wheat flour was determined using NIR hyperspectral imaging technology by Wang et al. (2018). From this study, it was found that the two wavelength bands with the largest differences between wheat flour and ADA were 1892 nm and 2039 nm, respectively, and the result showed that the minimum detected concentration of the optimal model was 0.2 g/kg. Additional applications of NIR spectroscopy in the detection of chemical additives in wheat flour are listed in Table 3.

**Table 3:** Applications of NIR spectroscopy in chemical additive detection of wheat flour.

Additives	Spectral range	Data analysis	Accuracy/Performance	Reference
talc content	400–2500 nm	RBF	R_p_ = 0.9999, RMSEP = 0.0765, RPD = 65.0909	Liu et al. (2019)
sodium hydroxymethanesulfonate	12500–4000 cm^−1^	LS-SVM	classification accuracy:92.0%–94.7%, detection limit = 1.5 mg/kg	Yuan et al. (2011)
azodicarbonamide	400–2498 nm	RBF	R = 0.99996, RMSEP = 0.5467, RPD = 116.5858	Che et al. (2017)
azodicarbonamide	400–2500 nm	RF	R^2^ = 0.99814, RMSEP = 2.91345, RPD = 23.54332	Du et al. (2021)
talcum powder or benzoyl peroxide (BPO)	900–1700 nm	two-band spectral analysis method	talcum powder and BPO powder under different depths of wheat flour were successfully detected	Fu et al. (2021)
benzoyl peroxide	1000–2500 nm	PLSR	R^2^_p_ = 1.000, SEP = 0.006%	Kim et al. (2022)
Talcum powder	12500–4000 cm^−1^	BP neural network	R^2^ = 0.9904, RMSEC = 0.8209, RMSEP = 1.8143	Liu et al. (2013)
Kojic acid	1000–2400 nm	PLS	R^2^ = 0.949–0.972, RMSE = 0.581%–0.830%, RPD = 4.171–4.830	Zhao et al. (2018)

### 5.2 Biological contamination

Biological contamination of wheat flour mainly includes deoxynivalenol (DON) and insects. DON also known as vomitoxin, is a major mycotoxin detected in wheat (Shen et al., 2022). DON contamination not only reduces wheat yield, but also causes vomiting, anorexia, teratogenicity, mutagenicity and carcinogenicity (Lippolis et al., 2014; Pestka, 2010). DON does not degrade easily, which threats wheat flour and the entire product chain (Wang et al., 2016). Although the contamination level of DON in wheat flour is relatively low, DON has distinct absorption in the near-infrared region (Peiris et al., 2009), and a number of studies have shown that it is feasible to measure DON in wheat samples with NIR spectroscopy (De Girolamo et al., 2009, 2014). In the study by Liang et al. (2020), the DON content of wheat flour samples was determined by SW-NIR reflectance spectroscopy, and the sparse autoencoder model yielded the highest prediction accuracy, with 100% for the training set and 96% for the test set. The dispersive NIR and Fourier transform NIR were applied to analyze 267 Brazilian wheat flour samples contaminated with DON by Tyska et al. (2021). The classification models of both partial least squares discriminant analysis and principal component analysis-linear discriminant analysis achieved accuracy rates of over 80%. Generally, whole wheat flour is made from intact wheat kernels, including the epidermis, which may be more susceptible to DON contamination (Zhang et al., 2019). T. Zhao et al. (2020) developed a scheme for the detection of DON contamination in whole wheat flour by Vis-NIR hyperspectral imaging, which can quickly analyze and ascertain whole wheat flour samples contaminated by DON. Wheat flour may also be contaminated by insects. Although NIR spectroscopy can quantitatively predict insect fragments in wheat flour to a certain extent, it cannot achieve high accuracy, and the sensitivity of NIR analysis needs to be further improved (Perez-Mendoza et al., 2003; Toews et al., 2007).

### 5.3 Flour adulteration in wheat flours

Wheat flours are divided into different varieties, and they have different uses and qualities. NIR spectroscopy combined with chemometric methods has been utilized to distinguish between common wheat flour and durum wheat flour (Unuvar et al., 2021). For example, einkorn is an old variety of wheat and is sold at higher prices than common wheat. Either to compensate for its weaker gluten structure or unfair economic profit, einkorn flour tends to be adulterated with bread wheat flour (Hidalgo et al., 2016). Ayvaz et al. (2021) assessed NIR spectroscopy to monitor bread wheat flour adulteration in einkorn flour and developed a PLSR calibration model for both flour mixtures. Highly accurate models yielded high R_p_ and RPD values of 0.99 and 19.3, respectively, and low SECV and SEP values of 1.12 and 1.39%, respectively. Furthermore, NIR spectroscopy was adopted to detect the adulteration of spelt flour with inexpensive bread wheat flour, and the resulting PLSR model achieved an R^2^ of 0.966 and an RMSEC of 5.2% (Ziegler et al., 2016).

Wheat flour is also susceptible to being adulterated or contaminated with inferior grains. For example, wheat flour may be mixed with some inexpensive grain flours, such as sorghum, corn and rice, which is very challenging to authenticate for consumers, especially when flours have a similar color. Verdú et al. (2016) developed a method for the detection of adulteration in wheat flour based on SW-NIR assisted by hyperspectral imaging technology. Taro flour in wheat flour was identified by the combination of near-infrared spectroscopy and multivariate analysis. Then, PCA was performed on the data, and the correct classification rate of the cross-validation model was 90.48% (Rachmawati et al., 2017). In another study by Su and Sun (2017), a predictive model using a spectral range of 900–1700 nm was established. The optimal model had the potential to authenticate the admixtures (common wheat flour, cassava flour and corn flour) in organic avatar wheat flour in the range of 3–75% (w/w). Additional applications of NIR spectroscopy for the detection of adulterated wheat flour are summarized in Table 4.

**Table 4:** Applications of NIR spectroscopy in adulterant detection of wheat flour.

Species	Spectral range	Data analysis	Accuracy/Performance	Reference
durum wheat flour, common bread wheat flour	400–2498 nm	PLS	sensibility of 0.5%	Marina et al. (2006)
cassava starch	1100–2500 nm	PLSR	certified additive-free wheat flour: r_pred_ = 0.977, RMSEP = 1.826 mg/kg, commercial wheat flour: r_pred_ = 0.995, RMSEP = 1.004 mg/kg,	De Almeida Duarte et al. (2022)
sorghum, oat and corn flours	400–1000 nm	MSPC	detection sensibility until 2.5%	Verdú et al. (2016)
potato flour	4000–10000 cm^−1^	PLS	R^2^_cv_ = 0.8865, RPD = 3.07	Wang et al. (2019)
peanut powder	935.61–1720.23 nm	PLSR	R^2^_p_ = 0.993–0.991, RMSEP = 0.251%–0.285%	Zhao et al. (2019)
unripe banana flour	447–1005 nm	PLSR	R^2^_c_ = 0.991; R^2^_p_ = 0.993; RPD = 12.021, RMSEC = 2.226 g/kg, RMSEP = 1.993 g/kg	Faith et al. (2019)
Chilean flour samples	1100–2000 nm	DPLS	correctly classified between 90% and 96%	González-Martín et al. (2014)
different origins and years wheat flour	950–1650 nm	LDA	correct percentages of 100% and 73%	Wadood et al. (2019)

## 6 Conclusions and future perspectives

Wheat flour is an important ingredient in food products, and ensuring its quality and safety is of great significance. Due to the advantages of being rapid, efficient and nondestructive, NIR spectroscopy has been shown to be an excellent technique for the quality and safety analysis of wheat flour. This review mainly reported recent advances in NIR nondestructive quality and safety analysis of wheat flour, including chemical composition, technological parameters, chemical additives, undesired contaminants and adulteration detection.

In general, NIR spectroscopy is a powerful tool for process analytical technology to assure the quality and safety of raw materials and final products. Therefore, online analysis of wheat flour is a future direction worth investigating. However, the application of NIR spectroscopy still faces some challenges due to the diversity of wheat flour samples and the complexity of NIR spectra data. Firstly, near-infrared models need to be updated according to the variability and differences of samples, and more suitable chemometric methods should be developed to maintain their predictive performance and improve the generalizability of the models. Secondly, low-cost and convenient NIR spectrometers are needed to promote the popularity of NIR spectroscopy technique in nondestructive analysis of wheat flour. Thirdly, the combination of NIR spectroscopy and other spectroscopic techniques (e.g. Raman and UV light) will broaden its application in wheat flour.

## CRediT authorship contribution statement

**Shun Zhang:** Conceptualization, and, Writing – original draft. **Shuliang Liu:** Writing – original draft, and, Writing – review & editing. **Li Shen:** Writing – original draft, and, Writing – review & editing. **Shujuan Chen:** Writing – original draft. **Li He:** Writing – original draft. **Aiping Liu:** Funding acquisition, Writing – review & editing, and, Supervision, All authors contributed to the article and approved the submitted version.

## Declaration of competing interest

The authors declare that they have no known competing financial interests or personal relationships that could have appeared to influence the work reported in this paper.
